# A comparative study of language learners’ ICT attitudes and behavior pre- and post-COVID-19

**DOI:** 10.1038/s41598-023-50872-0

**Published:** 2024-01-05

**Authors:** Anita Habók, Lan Anh Thuy Nguyen

**Affiliations:** 1 Institute of Education, University of Szeged, Szeged, 6722 Hungary; 2 MTA–SZTE Digital Learning Technologies Research Group, Szeged, 6722 Hungary; 3 Doctoral School of Education, University of Szeged, Szeged, 6722 Hungary

**Keywords:** Psychology, Mathematics and computing

## Abstract

Along with the adoption of hybrid teaching and learning modes, the integration of ICT into language instruction in higher education during the COVID-19 pandemic has afforded teachers and students increased opportunities to engage with technology. This quantitative study uses a self-report questionnaire to examine language learners’ attitudes and behavior tied to the role of ICT in learning before and after the COVID-19 pandemic. Firstly, the study confirms the questionnaire’s validity for assessing ICT attitudes and behavior in the Hungarian educational context. Additionally, the measurement invariance and differential item functioning of the assessment tool pre- and post-COVID-19 show that attitudes and the frequency of ICT use remain consistent at both the construct and item levels. Overall, the results demonstrate significant variations between the two student groups in attitudes and behavior tied to the use of ICT tools in language learning. The findings show that the post-COVID-19 group exhibits higher attitudes, and an increased frequency of technology use is observed compared to the pre-COVID-19 period. Furthermore, the study indicates that attitudes before COVID-19 do not strongly influence habitual technology use for language learning, while the opposite is true for the post-COVID-19 period.

## Introduction

The field of English as a foreign language (EFL) has widely employed information and communication technology (ICT) for various purposes in language learning and instruction. These digital tools and their multimedia elements have become an essential component of language teaching methods to accomplish learning objectives^1^. Furthermore, ICT has become more significant in language education as it enables educators to offer their students interactive and captivating learning experiences and facilitates self-directed learning and independence among learners. Digital technologies provide students with a secure and supportive environment to practice their language skills while also being a cost-effective means of delivering high-quality language instruction^2^. They can also help assess learners’ progress and provide real-time feedback, allowing teachers to adapt and customize their instruction to better suit their students’ needs.

In light of the positive effects of technology on language education, many studies have explored the factors linked to the implementation of digital resources within the educational setting (e.g.,^3^). Scientific research on ICT use within educational environments has observed that students’ attitudes or active involvement and control are crucial to the acceptance and effectiveness of ICT integration into the learning process^4^. The use of ICT in EFL classrooms may influence students’ learning journeys, since attitudes and behavior are considered influential factors in EFL learning. Several studies have also demonstrated the positive relation between learners’ positive attitudes toward technology and their success in EFL learning and using technological tools^5^. Furthermore, ICT can be employed to create captivating learning materials that would further stimulate students’ learning process. To effectively harness technology for language learning, stakeholders must consider students’ attitudes toward technology use^6^. Research has also observed a strong correlation between attitudes and technology use^7^, and it is worth noting that students’ attitudes toward ICT integration into language education can significantly affect their language proficiency^8^.

In Hungary, higher education institutions have been investing in digital facilities and incorporating technologies into the education system to support teachers and students. The COVID-19 pandemic in 2020 and 2021 further compelled Hungarian educational institutions to implement emergency teaching and learning measures to align with lockdown measures and strategies for mitigating COVID-19 transmission^9^. The transition from in-person instruction to remote teaching and learning was challenging for school stakeholders, especially for teachers and students, since digital education entailed a rapid adoption of new tools and platforms along with a radical rethinking of the learning process^10,11^. Despite these challenges, remote teaching allowed education to continue and helped limit the spread of the virus. Furthermore, online teaching provided teachers and students with more opportunities to integrate technology into their coursework. Indeed, during the pandemic, more opportunities to apply technology in learning may have changed students’ perceptions and ICT use in learning, enhancing their ability to address modern environmental challenges^12^. This change in attitude, if any, may result in the maintenance or acceleration of the digital transformation^13^, as students’ attitude toward ICT tools is a major driving factor in their acceptance of ICT and their behavior tied to the use of technology in education^14^. In the scientific literature, several investigations into students’ attitudes and behavior have been conducted before (e.g.^15^), and after the COVID-19 era (e.g.,^16^). However, the influence of COVID-19 on students’ attitudes and technology use has hardly been examined.

This study fills this gap by demonstrating changes in attitudes and behavior due to environmental concerns. Specifically, the study examines variations in Hungarian students’ attitudes and behavior tied to technology use in language learning between two periods: before and after COVID-19. Also, this research tests the reliability, validity, and invariance of an assessment tool between these two periods to guarantee the authenticity of the findings. Thus, four research questions have been formulated to investigate differences in students’ attitudes and ICT use in their language learning between the two study periods:

- RQ1. To what extent is the assessment tool valid for measuring students’ ICT attitudes and behavior in language learning?
- RQ2. To what extent is the assessment tool invariant pre- and post-COVID-19?
- RQ3. Are there significant differences between students’ ICT attitudes and behavior in the two periods?
- RQ4. Did the relation between students’ attitudes and behavior change during COVID-19?

## Theoretical background

For decades, scholars have investigated attitudes toward ICT in language education because of the advent of educational technology, since such attitudes shape teachers’ and students’ acceptance of the usefulness of ICT as well as the extent to which they incorporate technology into their language classrooms^17^. Studies have conceptualized attitudes toward ICT in several ways from specific to general. No universal attitude construct exists, and the manner in which attitudes are conceptualized varies across researchers, since attitude as a concept has been widely acknowledged among educational researchers to possess multiple dimensions and complexities^18^. Traditionally, attitudes toward technology use in language learning consist of cognitive, affective, and behavioral components^19^. First, the cognitive component of attitudes includes teachers’ or students’ knowledge or beliefs about the use of ICT in language learning. Second, the affective component entails their emotional perception and assessment of technology. Finally, the behavioral component refers to their ICT-related goals or activities.

Many scholars use these three conventional components of the attitude construct to measure students’ attitudes in the study environment, while others expand on them or reduce their number. For instance, Marshall and Cox^20^ covered all three conventional components of ICT attitude within EFL learning and instruction to examine students’ attitudes toward technology. Their scale incorporated certain factors: affective aspects, such as computer anxiety, affinity for technology, pleasure derived from using computers, and attitudes toward technology use within educational settings. They also considered cognitive aspects associated with the productivity and utility of technology and behavioral aspects of computer use for e-mails. Teo^21^ followed a different approach in constructing an attitude scale, administering a questionnaire with three scales—enjoyment, importance, and anxiety. These three attitude elements were divided into affective (enjoyment and anxiety) and cognitive (importance). The exclusion of certain classic components of attitude may be attributed to varying perceptions regarding their significance. Variations in the use of distinct subscales to measure attitude may be attributed to the influence of cultural research contexts and researchers’ psychological perspectives.

The three classical aspects of attitudes have served as a scaffold for expanding constructs in multiple educational research cultures across periods (e.g.,^1,22–29^, and the three primary components of attitude can influence each other^30^. To illustrate, Kearney et al.^27^ developed an attitude assessment tool composed of five distinct aspects: behavioral engagement, technology confidence, English confidence, emotional engagement, and attitude toward incorporating technology in English language teaching. Notably, the inclusion of subject-specific content on the attitude subscales aimed to accurately discern students’ attitudes toward learning English using technology. Nagy and Habók^25^ devised and validated a questionnaire that included both internal and external components to evaluate students’ attitudes toward ICT within the context of Hungarian EFL education. This questionnaire was rooted in the classical dimensions of the three-component model, which supported various factors. Furthermore, the authors expanded upon and established connections between the fundamental elements and other aspects pertinent to the modern language classroom. Similarly, Aryadoust et al.^31^ extensively developed and validated a three-component ICT attitude instrument for Iranian EFL learners, which aimed to measure their attitudes toward using technology in their English language learning, encompassing behavioral, affective, and language skill components.

Previous empirical studies suggest that attitudes toward ICT correlate with technology knowledge and experience^32^. Positive attitudes increase technology use, highlighting the connection between attitude and behavior^14^. Studies across different EFL contexts, such as Alothman et al.^33^, show positive attitudes but limited adoption of ICT in practice. Despite favorable attitudes, actual implementation of ICT in academic settings remains restricted, as seen in studies like Park and Son^34^ and Nguyen and Habók^2^. Online learning during the COVID-19 pandemic has shown benefits for motivation, anxiety levels, and overall attitude toward language learning^35^. However, unfavorable attitudes toward technology may contribute to limited adoption in practice^36^.

## Results

### RQ1. To what extent is the assessment tool valid for measuring students’ ICT attitudes and behavior in language learning?

**Figure 1:**
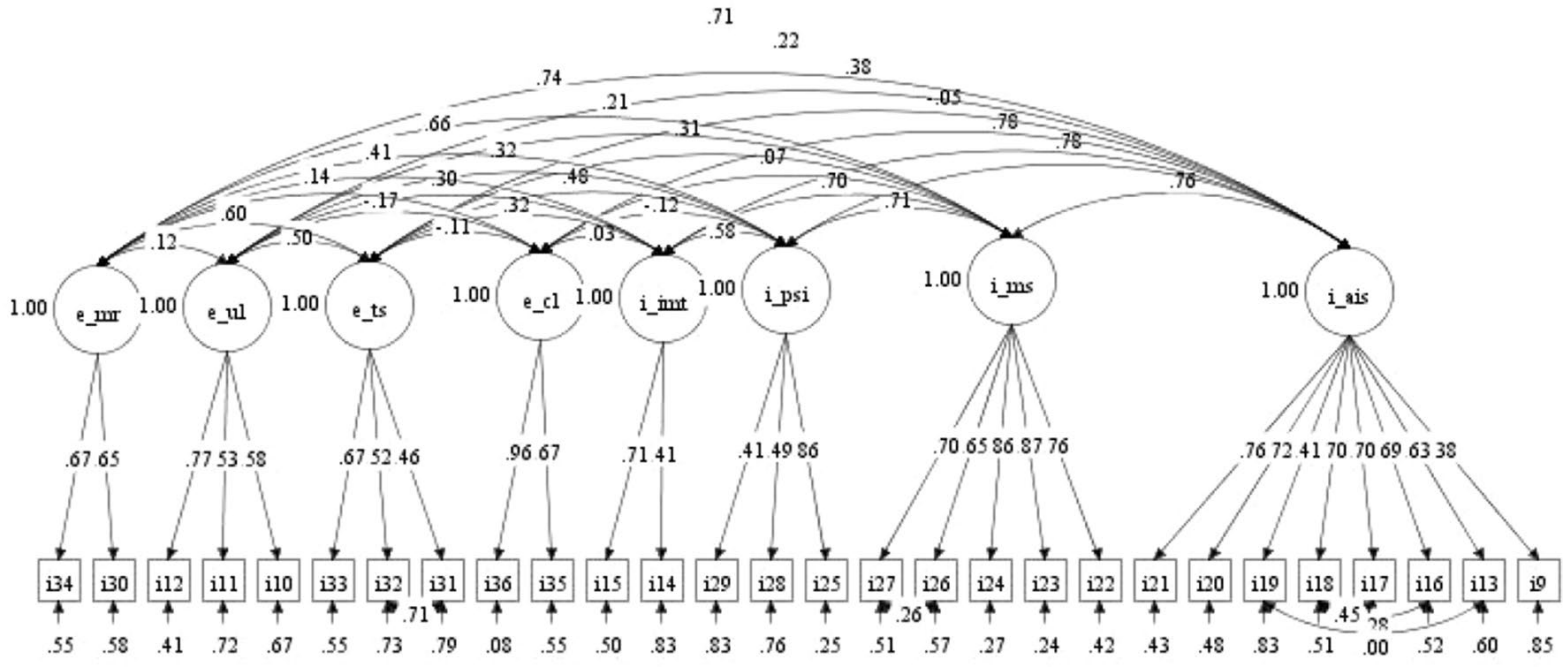
Confirmatory factor analysis model of the attitude scale. *Note*. I_AIS: affective ICT strategies; I_MS: metacognitive strategies; I_PSI: personal significance of ICT; I_IMT: importance of mobile tools; E_CL: curriculum-based limitations; E_TS: task-centered strategies; E_UL: use of ICT tools in learning; E_MR: motivating role of ICT.

The first step involved testing a multidimensional CFA model to evaluate whether the measurement model fit the data for the entire sample. For the attitude scale, after some error pairs in the same factors were connected, the data showed a good model fit (χ^2^ [317] = 909.37, *p* < 0.001, CFI = 0.911, RMSEA = 0.073, 90% CI RMSEA = [0.068, 0.079], SRMR = 0.063) (Fig. 1). With regard to the questionnaire’s reliability, all factors achieved good or acceptable Cronbach’s alpha values from 0.50 to 0.88. The internal metacognitive strategies subscale obtained the highest values, while the internal importance of mobile tools subscale had the lowest. Despite the low Cronbach’s alpha value for internal importance of mobile tools, the entire attitude scale’s internal consistency reliability was acceptable, with a total Cronbach’s alpha value of 0.89 (Table 1). In addition, although the attitude subscales had some low AVE and CR values, those for the total attitude scale were acceptable with a CR value of above 0.60^37^ (Table 2).

**Table 1:** Reliability of the attitude scale.

Attitude	Cronbach’s alpha	Number of items	AVE	CR
I_AIS	0.85	8	0.40	0.84
I_MS	0.88	5	0.59	0.87
I_PSI	0.60	3	0.38	0.62
I_IMT	0.50	2	0.33	0.48
E_CL	0.78	2	0.68	0.80
E_TS	0.76	3	0.31	0.56
E_UL	0.65	3	0.39	0.65
E_MR	0.65	2	0.43	0.60
Attitude	0.89	28	0.44	0.95

**Table 2:** Discriminant validity of the attitude scale.

	I_AIS	I_MS	I_PSI	I_IMT	E_CL	E_TS	E_UL	E_MR
I_AIS	**0.632**							
I_MS	0.707**	**0.768**						
I_PSI	0.549**	0.541**	**0.616**					
I_IMT	0.530**	0.492**	0.320**	**0.574**				
E_CL	− 0.027	0.067	− 0.082	0.041	**0.824**			
E_TS	0.259**	0.271**	0.256**	0.197**	− 0.007	**0.556**		
E_UL	0.191**	0.152**	0.268**	0.106*	− 0.123*	0.270**	**0.624**	
E_MR	0.528**	0.573**	0.423**	0.268**	0.048	0.303**	0.046	**0.655**

The square root of average variance extracted values are in bold.

Component correlation matrix (Pearson correlation).

*The correlation achieved statistical significance at the 0.05 level (2-tailed).

**The correlation achieved statistical significance at the 0.01 level (2-tailed).

**Figure 2:**
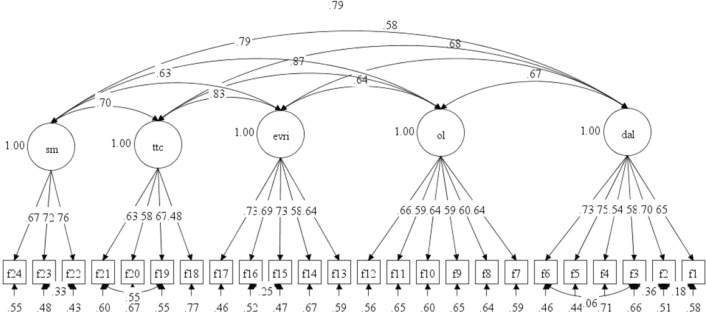
Confirmatory factor analysis model of the behavior scale. *Note.* SM: social media; TTC: task-based tools and communication; EVRI: editing and visual representation of information; OL: online learning; DaL: dictionaries and lexicon.

As regards frequency of ICT use, five items were deleted to guarantee data fit. After connecting some pairs of errors belonging to the same factor, the measurement model achieved an acceptable fit to measure students’ behavior tied to technology implementation in their language learning (χ^2^ [236] = 676.63, *p* < 0.001, CFI = 0.900, RMSEA = 0.073, 90% CI RMSEA = [0.067, 0.080], SRMR = 0.061) (Fig. 2). Meanwhile, with respect to the frequency of using ICT tools, reliability values ranged from 0.70 to 0.79, and the total scale achieved good reliability at 0.90 (Table 3). Some AVE values for the subscales were below 0.50, but all CR values were above 0.60, confirming the convergent validity of the frequency of ICT use.

**Table 3:** Reliability of the behavior scale.

Frequency of use (behavior)	Cronbach’s alpha	Number of items	AVE	CR
DaL	0.78	6	0.43	0.82
OL	0.77	6	0.38	0.78
EVRI	0.79	5	0.45	0.80
TTC	0.70	4	0.39	0.66
SM	0.77	3	0.51	0.76
Frequency of use	0.90	24	0.43	0.94

Furthermore, the square root of the AVE and the interconstruct correlation in the component correlation matrix were compared to assess the discriminant validity of the attitude and frequency of ICT use scales. The models’ discriminant validity was confirmed, as the square roots of the AVE surpassed the correlation values observed between the different factors (Tables 2 and 4).

**Table 4:** Discriminant validity of the frequency of ICT use scale.

	DaL	OL	EVRI	TTC	SM
DaL	**0.655**				
OL	0.464**	**0.616**			
EVRI	0.448**	0.433**	**0.670**		
TTC	0.309**	0.586**	0.547**	**0.624**	
SM	0.476**	0.559**	0.395**	0.442**	**0.714**

The square root of average variance extracted values are in bold.

Component correlation matrix (Pearson correlation).

**The correlation achieved statistical significance at the 0.01 level (2-tailed).

### RQ2. To what extent is the assessment tool invariant pre- and post-COVID-19?

After confirming the validity of the measurement tools for the entire sample, we performed measurement invariance analysis using multigroup CFA to test whether the questionnaires are invariant pre- and post-COVID-19. We tested the measurement model for configural, metric, and scalar invariance by systematically imposing equality constraints on the factor loadings and item intercepts across two distinct groups: pre-COVID-19 and post-COVID-19. The configural invariance model assumes an equivalent number of latent variables (factors) and item-factor associations (items loading on the same factor in both groups) across two groups with all model parameters estimated independently for each group. Metric invariance equates the latent variables based on equal loadings across groups. Scalar invariance models are based on equality constraints on item intercepts (means of item responses). Relating two groups with this invariance level enables a comparison of factor means. In this case, when such level of invariance is achieved, it becomes justifiable to compare factor means between the two groups^38^.

For the grouping variable, pre- and post-COVID-19, the configural model displayed a good data fit. These results support the suitability of the nested factor model for both groups. Moreover, the metric and scalar invariance models exhibited satisfactory fit values. The psychometric literature has proposed different threshold values for changes in CFI, RMSEA, and SRMR. To illustrate, Chen^39^ suggested that model fit changes could be considered nonsignificant when ΔCFI ≤  − 0.010, ΔRMSEA ≤ 0.015, and ΔSRMR ≤ 0.030. As regards model fit changes in the multigroup CFA, the criteria for ΔCFI, ΔRMSEA, and ΔSRMR values were all satisfied (Tables 5 and 6). This indicates that the attitude and ICT use frequency measurements were invariant and consistent in evaluating students’ attitudes and behavior tied to technology use in language learning pre- and post-COVID-19. This finding confirms the validity and reliability of comparing means between groups and suggests that the same scale can be used to measure the same construct across the two groups, thus allowing for a more comprehensive assessment of the construct as well as a more accurate comparison between different groups^40^.

**Table 5:** Goodness-of-fit statistics and multigroup invariance attitude comparisons (grouping variable: pre- and post-COVID-19).

Measurement invariance models	χ^2^	df	CFI	RMSEA [90% CI]	SRMR	Δ χ^2^ (Δ2 (df))	ΔCFI	ΔRMSEA	ΔSRMR
Model 1: Configural	1261.66	634	0.910	0.075 [0.069, 0.081]	0.073	–	–	–	–
Model 2: Metric	1289.99	654	0.906	0.075 [0.069, 0.081]	0.075	28.33 (20)	− 0.004	0.000	0.002
Model 3: Scalar	1367.19	674	0.900	0.077 [0.071, 0.083]	0.080	77.20 (20)	− 0.006	0.002	0.005

**Table 6:** Goodness-of-fit statistics and multigroup invariance behavior comparisons (grouping variable: pre- and post-COVID-19).

Measurement invariance models	χ^2^	df	CFI	RMSEA [90% CI]	SRMR	Δ χ^2^ (Δ2 (df))	ΔCFI	ΔRMSEA	ΔSRMR
Model 1: Configural	1027.88	472	0.901	0.079 [0.073, 0.085]	0.073	–	–	–	–
Model 2: Metric	1072.73	491	0.896	0.079 [0.073, 0.085]	0.079	44.85 (19)	− 0.005	0.000	0.006
Model 3: Scalar	1128.89	510	0.899	0.080 [0.074, 0.086]	0.079	56.16 (19)	0.003	0.001	0.000

After comparing measurement invariance between the two groups at the construct level, we addressed measurement invariance concerns at the item level, which is commonly achieved through DIF methods^41^. The items for the attitude and ICT use scales were unbiased between the pre- and post-COVID-19 groups when DIF values aligned with prescribed benchmarks: (1) insignificantly biased if DIF ≤ 0.43 logits, (2) slight to moderate bias if DIF ≥ 0.43 logits, and (3) moderate to substantial bias if DIF ≥ 0.64 logits^42^.

**Figure 3:**
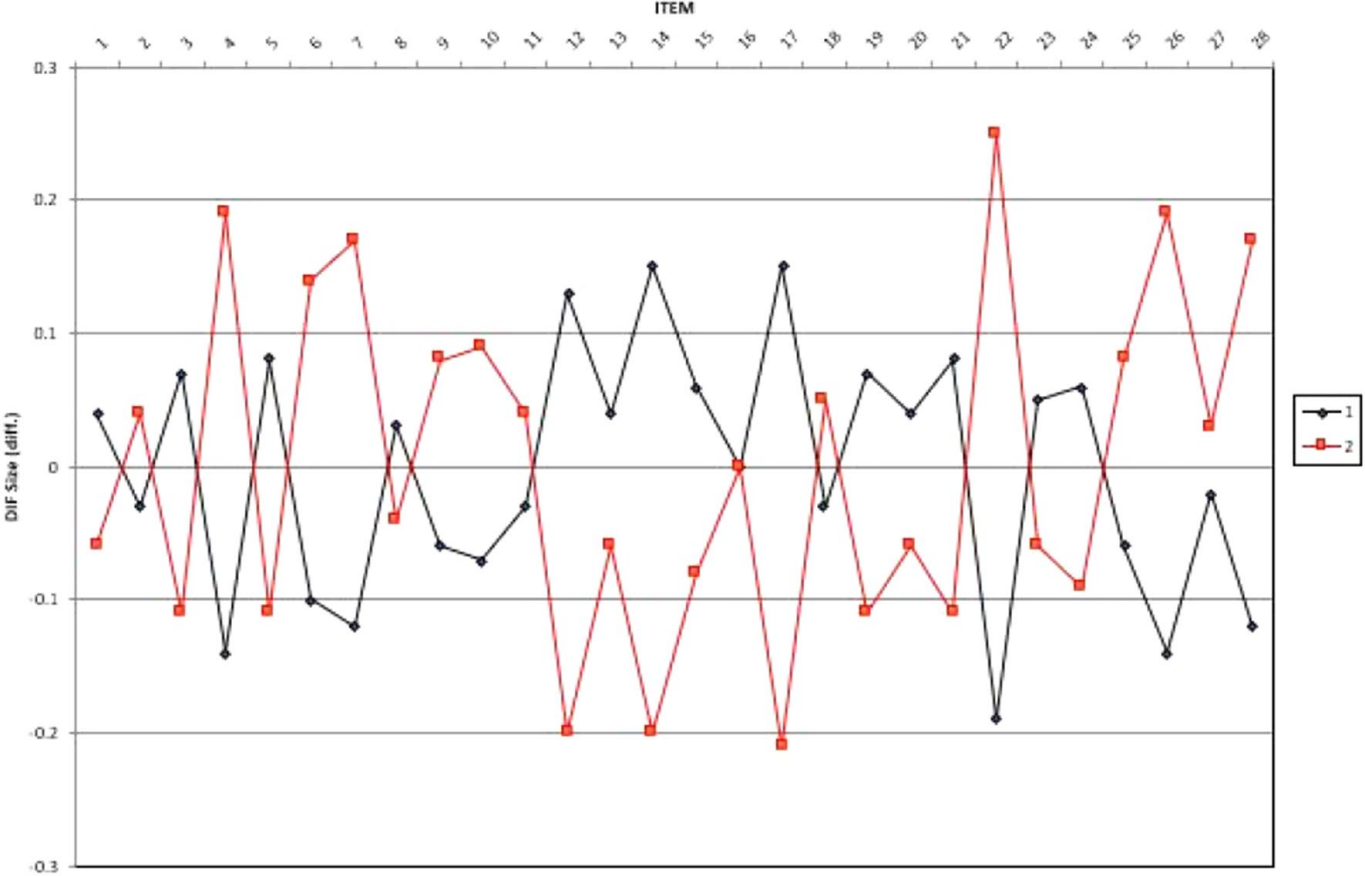
Differential item functioning size of the attitude scale.

**Figure 4:**
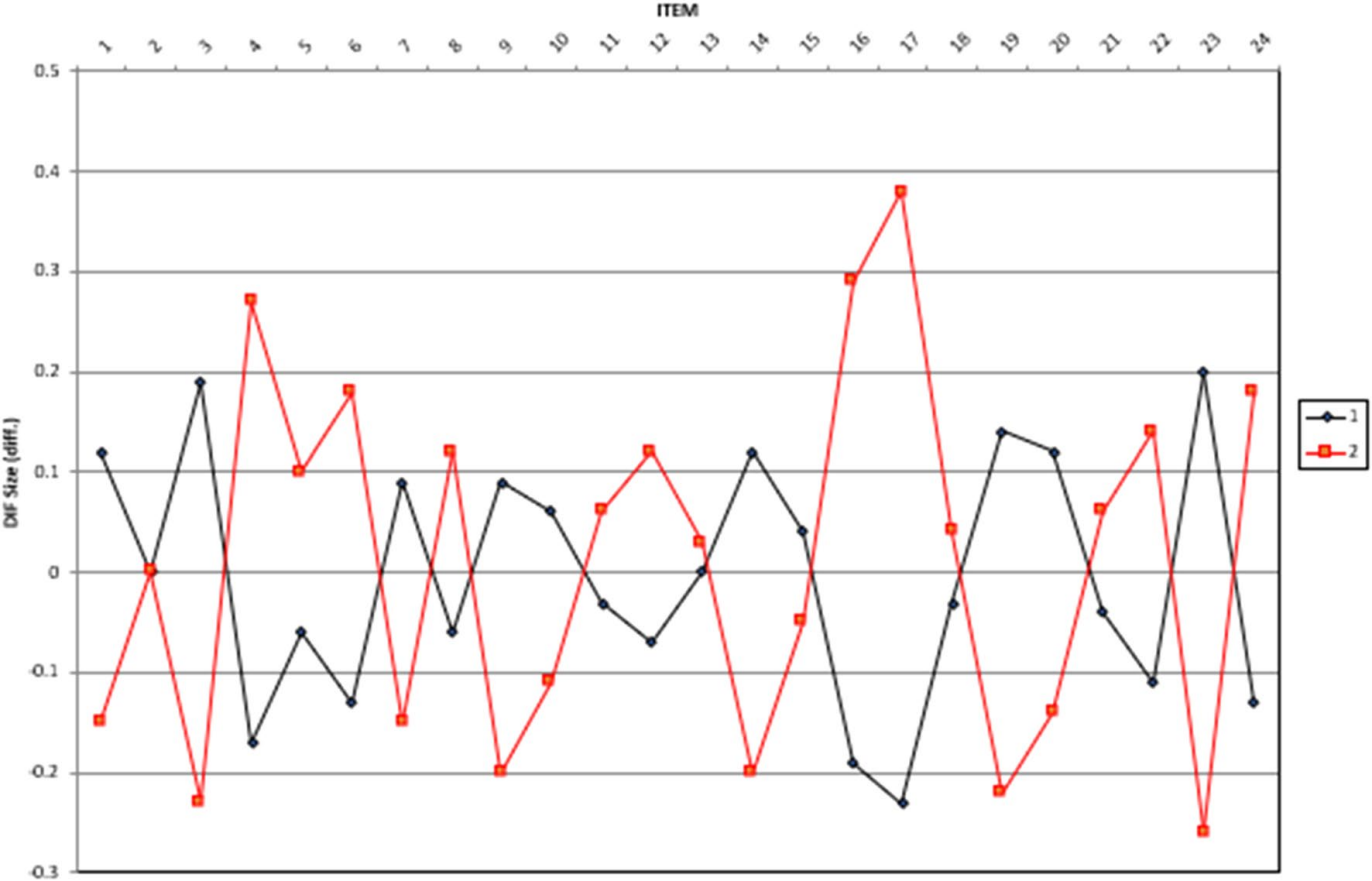
Differential item functioning size of the behavior scale.

DIF analysis showed that out of the total number of items on the attitude and behavior (frequency of use) scales, 28 and 24 items, respectively, were classified as insignificantly biased (Figs. 3 and 4). Specifically, differential size range varied from − 0.21 to 0.25 for attitude items and from − 0.26 to 0.38 for behavior items. These findings suggest a consistent DIF size between the two groups on both the attitude and behavior scales. Consequently, further investigation can be pursued with confidence. In sum, at the construct and item levels, attitude and technology use frequency are invariant between the two groups, and the data could be investigated for further comparison.

### RQ3. Are there significant differences between students’ ICT attitudes and behavior in the two periods?

After testing the invariance of the measurement tools across the two periods, we performed latent mean analysis to measure disparities in Hungarian language learners’ attitudes and behavior tied to ICT tools. After comparing latent means between the groups pre- and post-COVID-19, the former was considered as the reference group. By fixing the latent mean differences in the pre-COVID-19 period to zero, the latent means for the other group indicated differences between the two^43^. As regards students’ attitudes toward ICT use in their language education, the results of the latent mean differences indicated statistically significant distinctions between the two student groups. Specifically, students who filled out the questionnaire post-COVID-19 had higher latent means than those who did the same before the pandemic, suggesting that students were more positive toward the implementation of technologies in their language learning after experiencing the pandemic. The means for all the attitude scale components for the post-COVID-19 group were higher than those for the pre-COVID-19 group except for two subscales: external curriculum-based limitations and external motivating role of ICT. Differences between the two groups were insignificant among all the subscales (*p* > 0.005) except for internal importance of mobile tools and external use of ICT tools in learning. Table 7 shows the latent mean differences between the two groups.

**Table 7:** Latent mean differences between the two groups for attitude toward ICT tools.

Variable	Attitude	I_AIS	I_LMS	I_PSI	I_IMT	E_CL	E_TS	E_UL	E_ML
Mean Difference	0.142	0.028	0.137	0.292	0.267	− 0.010	0.037	0.529	− 0.142
*p*	0.000	0.185	0.116	0.008	0.000	0.926	0.596	0.000	0.110
SD	1.414	0.391	1.622	2.052	1.137	1.977	1.287	1.193	1.660
Cohen’s *d*	0.100	0.071	0.084	0.142	0.234	− 0.005	0.028	0.443	0.085

As regards the frequency of ICT use among Hungarian students in their language learning, a notable disparity was observed between the two groups (*p* < 0.001), with students using technologies in their language learning more frequently after the pandemic than beforehand. The mean differences between the two groups were significant on all the subscales except for editing and visual representation of information (Table 8). Among the ICT tool components, Hungarian students showed a trend of using social media in their language learning much more frequently than other components post-COVID-19 (z = 0.809).

**Table 8:** Latent mean differences for frequency of ICT use between the two groups.

Variable	Frequency	DaL	OL	EVRI	TTC	SM
Mean Difference	0.321	0.332	0.275	0.036	0.157	0.809
*p*	0.000	0.000	0.000	0.641	0.000	0.000
SD	1.413	1.641	1.156	1.417	0.783	2.070
Cohen’s *d*	0.227	0.202	0.237	0.025	0.200	0.390

To ensure a standardized measurement of effect size, studies have proposed using Cohen’s *d* indices^44^. First, the variances between two groups must be assumed to be equal. Cohen’s *d* coefficients can then be calculated once the shared standard deviations of both groups are satisfied^43^. The Cohen’s *d* indices in Tables 7 and 8 indicate small effect size values for attitude toward ICT tools (*d* = 0.100) and frequency of ICT use in language learning (*d* = 0.227). This means that although students’ attitudes toward ICT were more positive and they used technologies in their language learning more frequently post-COVID-19, the effect size of these changes was not large.

### RQ4. Did the relation between students’ attitudes and behavior change during COVID-19?

**Figure 5:**
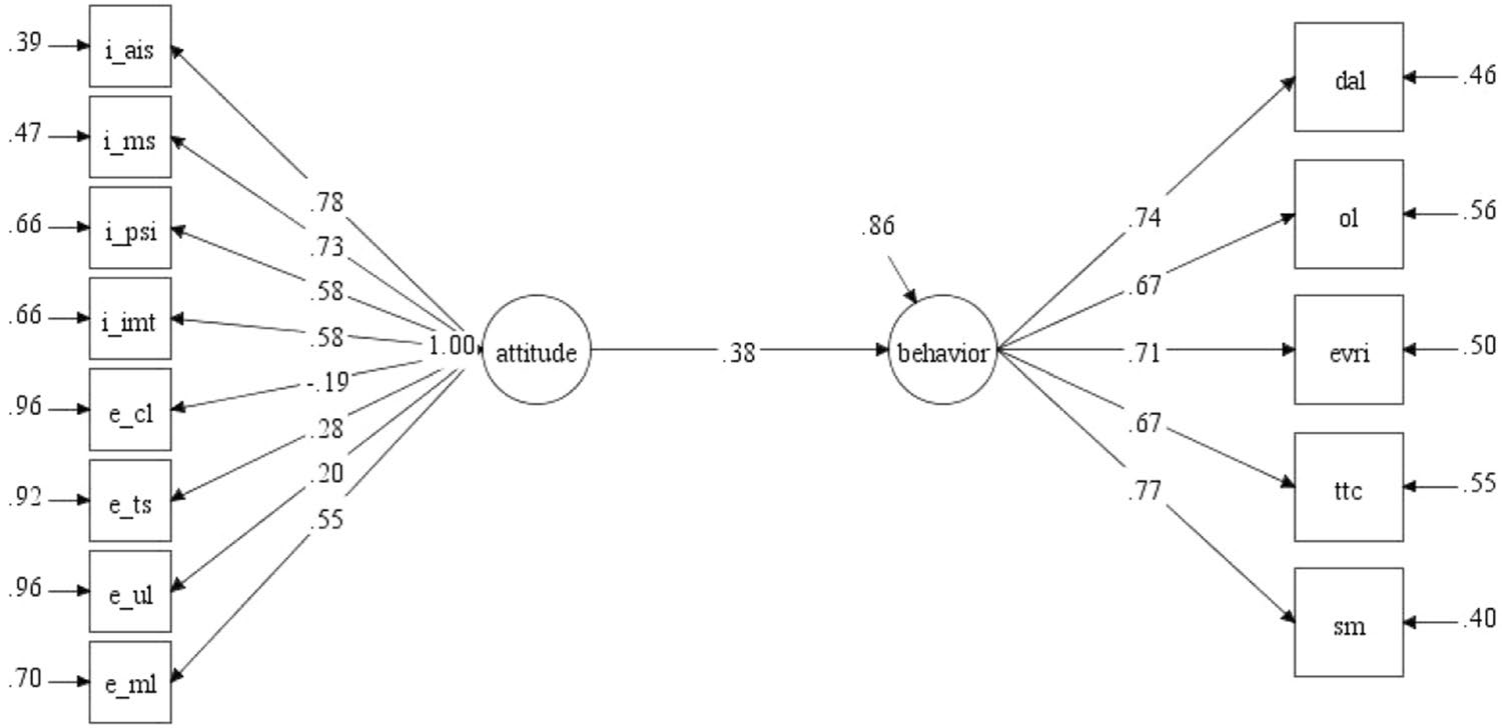
Relation between attitude and behavior (pre-COVID-19).

**Figure 6:**
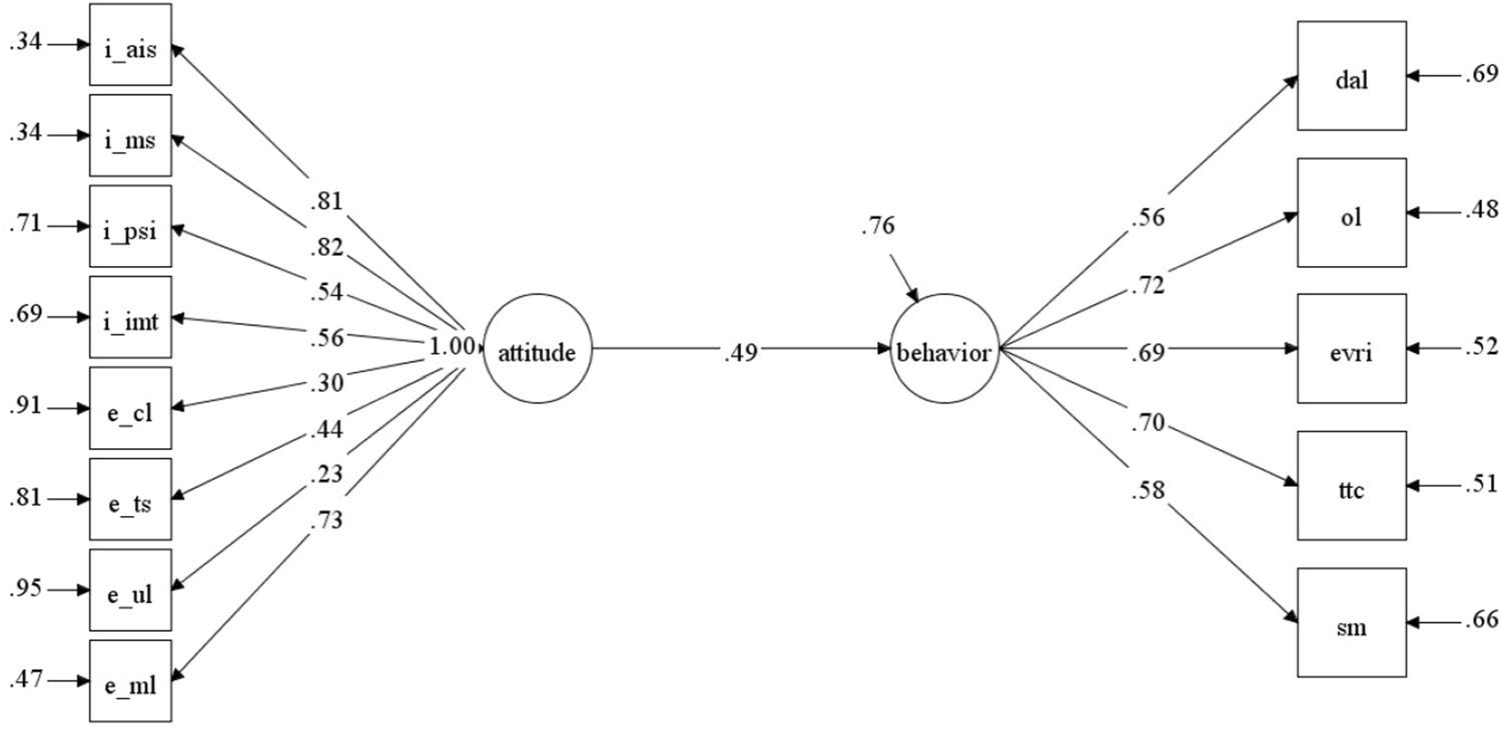
Relation between attitude and behavior (post-COVID-19).

To investigate the correlation between students’ ICT attitudes and behavior, we analyzed two structural equation models that measure the relation between attitude and behavior (Figs. 5 and 6).

These models showed an acceptable fit (before COVID-19) and a good fit (after COVID-19) with two data groups: the pre-COVID-19 model: χ^2^ [64] = 161.23, *p* < 0.001, CFI = 0.900, RMSEA [90% CI] = 0.074 [0.060, 0.088], SRMR = 0.062; the post-COVID-19 model: χ^2^ [64] = 87.96, *p* < 0.001, CFI = 0.954, RMSEA [90% CI] = 0.050 [0.019, 0.075], SRMR = 0.057. The relation between the two variables was observed to have changed across these two periods. Specifically, students’ behavior tied to ICT use in classroom settings can be predicted from their attitudes better before COVID-19 than afterward (β_pre-Covid_ = 0.38, *p* < 0.01; β_post-Covid_ = 0.49, *p* < 0.01). As a result, students’ attitudes did not affect their frequent use of technology in language learning as much as they did after COVID-19.

## Discussion and conclusion

The main objective of this study was to examine changes in Hungarian students’ attitudes and behavior tied to the integration of technology into their language learning. This was achieved by administering a questionnaire composed of two main sections: attitudes toward ICT tools and frequency of ICT use. Although several instruments have been developed or modified to measure EFL learners’ attitudes and behavior tied to ICT tools across different educational contexts and periods, studies have yet to offer a well-structured validity argument for these measures^31^. Hence, this study established the reliability and validity of a measurement tool within the Hungarian higher education setting as an initial step toward confirming the validity of subsequent analysis findings. Overall, the instrument demonstrated reliability and validity within the Hungarian research context for measuring language learners’ attitudes toward ICT tools and behavior associated with ICT integration in language education. This study then used the same questionnaire to compare students’ ICT attitudes and behavior in two different periods (pre- and post-COVID-19). To ensure the consistency of measurement across these periods, the questionnaire’s measurement invariance was tested for both groups. Multigroup CFA results indicated the absence of noteworthy changes in model fit for the configural, metric, and scalar models, suggesting that the measurements were invariant and consistent at the construct level in assessing students’ attitudes and behavior tied to technology use in their language education both before and after COVID-19. This study also examined item-level DIF, detecting no bias among the assessment items between the pre- and post-COVID-19 groups. In general, the findings of the study show an improvement in students’ attitudes and behavior toward ICT amid the COVID-19 pandemic. Moreover, the correlation between students’ attitudes and their behavior in using technology for learning appears to be more precise, especially in the post-COVID-19 era.

Students’ attitudes toward ICT improved during the COVID-19 pandemic, a finding which is consistent with those of other studies (e.g.,^35^). Additionally, the mean scores for the post-COVID-19 group for all attitude scale components were higher than those for the pre-COVID-19 group except for two subscales: external curriculum-based limitations and external motivating role of ICT. The higher attitude levels observed in the pre-COVID-19 group for these components may be attributed to the curriculum design, which was initially intended for in-person or hybrid education. The sudden emergence of the COVID-19 outbreak compelled the Hungarian education system to switch to a fully online teaching and learning format with no preparations in the curriculum or digital competencies among teachers or students. During COVID-19, students may have increased their awareness of the limitations of the curriculum when applied to fully online learning as well as their insufficient digital proficiency, which posed challenges for both teachers and students during the pandemic. Moreover, the findings also demonstrated negligible variances in attitude means between the two groups across all the subscales except for internal importance of mobile tools and external use of ICT tools in learning. This result can be attributed to the crucial role of digital devices in ensuring access to online education during the emergency teaching and learning period. Moreover, the complete integration of technology into language education during the pandemic was vital for students to acquire their desired language knowledge and skills. With respect to behavior tied to ICT use in language learning, students reported a higher frequency of technology use after the pandemic period compared with their previous usage, a finding which could be because they had become more familiar with the technology and had had more time and opportunities to adapt to it during the pandemic. Furthermore, in terms of the different components of ICT tools, Hungarian students were significantly inclined to use social media platforms in their language learning to a significantly higher extent compared to other components post-COVID-19. This finding is consistent with other recommendations on the efficacy and collaborative nature of online learning during the pandemic (e.g.,^45^). Therefore, students are more likely to use technology to continue their language learning and may be more aware that technology use has become increasingly necessary to keep up with their studies^46,47^. Results also showed that despite the minor impact of the COVID-19 pandemic on students’ ICT attitudes and behavior, higher education stakeholders in Hungary must nevertheless identify language education strategies to address uncertainty after the pandemic. For instance, Rasli et al.^48^ proposed several approaches for higher education institutions to address ambiguity and unpredictability in resilience and change management, digital transformation and online learning, curriculum modification, and sustainability. Notably, the study’s experts extensively deliberated on the significance of flexibility as a prevailing concern.

With regard to changes in the correlation between Hungarian students’ ICT attitudes and behavior in language learning, this study confirmed other findings that have established a significant relation between these variables^14,49^. This suggests that students’ behavior tied to using technology in their learning can be predicted more accurately based on their attitudes, particularly post-COVID-19. Despite only experiencing a temporary shift to remote education during the COVID-19 pandemic, students have recognized the benefits of technology in their language education, resulting in a more positive influence of their ICT attitudes on their behavior tied to technology integration and better overall performance in the subject^10^. These findings indicate that when students embrace technology with a positive mindset, they improve their language proficiency, highlighting the benefit of favorable attitudes in successful language learning. Therefore, when introducing technology in the classroom, educators should strive to cultivate a positive environment as well as guarantee the provision of essential resources to students. Educators must also actively encourage students to incorporate technology into their language learning process^46^. This approach will increase students’ engagement, build their confidence in their technological abilities, and equip them with the required skills to thrive in an increasingly technology-driven world. At a deeper level, higher education institutions must consider changing the curriculum to promote online learning and address uncertainty. Additionally, it is important to improve the education system with adaptable teaching and learning methods, enhance teachers’ and students’ digital literacy through training, offer school stakeholders effective, state-of-the-art digital facilities in educational institutions, and recommend workable solutions for any potential unknowns.

The study has several limitations. First, low values for Cronbach’s alpha, AVE, and CR suggest potential reliability issues. Furthermore, it is necessary to address interconnected errors to achieve satisfactory or desirable model fitness indices. However, these issues do not impact the total reliability and validity of the questionnaire, as the overall reliability values for the attitude scale and the frequency of use scale are satisfactory. As regards model fitness, connecting error pairs in the same factors with similar characteristics is a common method in structural equation modeling studies. Future research could focus on comprehensively revising questionnaire items or measurement scales to enhance their reliability and validity. Additionally, this study employed a quantitative self-report design, which may introduce bias between self-appraisal and actual attitudes and behavior, providing the opportunity for future research to adopt a mixed-methods approach for a better understanding of students’ attitudes and ICT use in language learning. Moreover, since this study recruited a small sample of EFL learners in Hungary, resulting in the findings not being generalizable to other language education contexts, future studies may incorporate larger samples from different regions in the country to improve the external validity of the findings.

## Methods

### Participants

The sample consisted of undergraduate students from a university in Hungary, representing various majors across different faculties. Approximately 90% of the participants were enrolled in teacher education programs, while the remaining 10% were pursuing academic disciplines in the humanities and sciences. The measurement involved 348 students, with 200 participants (N_male_ = 82, N_female_ = 118) completing the instrument before the COVID-19 pandemic and 148 of them (N_male_ = 50, N_female_ = 98) responding to the questionnaire after COVID-19. With reference to English language proficiency levels, as outlined by the Common European Framework of Reference for Languages, half of the participants (174 students) exhibited proficiency at the upper-intermediate level. Furthermore, 23% of the students attained an advanced level, and 14% achieved an intermediate level. Conversely, a smaller proportion of participants demonstrated proficiency at the advanced and elementary levels, comprising 3% and 4%, respectively, while 6% were at the beginner level.

### Instrument

This study uses a self-report questionnaire to evaluate university students’ attitudes and behavior tied to ICT use in their English language learning^25^, the questionnaire has been validated in a Hungarian educational context^50^. The attitude scale distinguishes between internal and external domains: the internal domain is comprised of affective ICT strategies (I_AIS), metacognitive strategies (I_MS), personal significance of ICT (I_PSI), and importance of mobile tools (I_IMT), while the external domain consists of curriculum-based limitations (E_CL), task-centered strategies (E_TS), use of ICT tools in learning (E_UL), and the motivating role of ICT factors (E_MR). A sample item is “Using a computer for learning makes me happy.” The behavior field, which examines the frequency at which students use a tool or perform an activity, is made up of the following factors: dictionaries and lexicon, online learning, editing and visual representation of information, task-based tools and communication, and social media. In this case, we listed the tools, and the participants rated the frequency of their use. Within both domains, the students used a four-point rating scale to provide their responses. For attitude-related questions, the scale ranged from disagreement to agreement, and, for behavior-related questions, the scale ranged from infrequent to frequent occurrences, capturing the regularity of specific actions or behavior.

### Procedure

According to the IRB at the Doctoral School of Education, University of Szeged, this study met the necessary ethical requirements and could be conducted. After permission was granted for the study, the participants were informed about the research purpose, that their data would be treated with anonymity, and that their responses would be kept confidential with no disclosure to external parties. After agreeing to participate, the students completed the questionnaire online in Hungarian, which took approximately 20 min, and they did so at a time that was most convenient for them. The researcher who collected the data was available to answer any questions, but the respondents did not comment on the completion of the questionnaire or any technical issues.

### Data analysis

First, we analyzed the questionnaire’s factor structure for both groups using confirmatory factor analysis (CFA) fit indices, such as the comparative fit index (CFI), root mean square error of approximation (RMSEA), standardized root mean square residual (SRMR), and chi-square (χ^2^) value. Additionally, we assessed the tool’s convergent validity, including Cronbach’s alpha value, average variance extracted (AVE), and composite reliability (CR) as well as its discriminant validity. Second, we performed multigroup CFA to analyze the invariance of the assessment tool construct and used differential item functioning (DIF) methods to test the items. Third, we investigated mean differences *and* Cohen’s *d* to explore participants’ attitudes and behavior before and after COVID-19. Fourth, we employed structural equation modeling to ascertain the correlation between attitude and behavior, using fit indices for assessment.

## Data Availability

The researchers assured the participants that their responses would be treated confidentially and would not be disclosed to third parties. Therefore, the dataset used for this study can be shared with the specific permission from the corresponding author upon reasonable request.
